# Epstein-Barr Virus but Not Cytomegalovirus Is Associated with Reduced Vaccine Antibody Responses in Gambian Infants

**DOI:** 10.1371/journal.pone.0014013

**Published:** 2010-11-17

**Authors:** Beth Holder, David J. C. Miles, Steve Kaye, Sarah Crozier, Nuredin Ibrahim Mohammed, Nancy O. Duah, Elishia Roberts, Olubukola Ojuola, Melba S. Palmero, Ebrima S. Touray, Pauline Waight, Matthew Cotten, Sarah Rowland-Jones, Marianne van der Sande, Hilton Whittle

**Affiliations:** 1 MRC Laboratories Gambia, Banjul, The Gambia; 2 South African Tuberculosis Vaccine Initiative, Institute of Infectious Disease and Molecular Medicine, University of Cape Town, Cape Town, South Africa; 3 Jefferiss Trust Laboratory, Imperial College London, London, United Kingdom; 4 MRC Epidemiology Resource Centre, University of Southampton, Southampton General Hospital, Southampton, United Kingdom; 5 London School of Hygiene & Tropical Medicine, London, United Kingdom; 6 Immunisation Department, Health Protection Agency Centre for Infections, London, United Kingdom; 7 Centre Infectious Disease Control (CIb), National Institute for Public Health and the Environment (RIVM), Bilthoven, The Netherlands; 8 Julius Center, University Medical Center Utrecht (UMCU), Utrecht, The Netherlands; University of Toronto, Canada

## Abstract

**Background:**

Epstein-Barr virus (EBV) and cytomegalovirus (CMV) are persistent herpesviruses that have various immunomodulatory effects on their hosts. Both viruses are usually acquired in infancy in Sub-Saharan Africa, a region where childhood vaccines are less effective than in high income settings. To establish whether there is an association between these two observations, we tested the hypothesis that infection with one or both viruses modulate antibody responses to the T-cell independent meningococcal polysaccharide vaccine and the T-cell dependent measles vaccines.

**Methodology/Principal Findings:**

Infection with EBV and CMV was diagnosed by the presence of virus-specific IgM in the peripheral blood or by the presence of IgG at higher levels than that found in umbilical cord blood. Anti-meningococcus IgG and IgM were quantified by ELISA. Anti-measles antibody responses were quantified by haemagglutinin antibody inhibition assay. Infants infected with EBV had reduced IgG and IgM antibody responses to meningococcal polysaccharides and to measles vaccine. Infection with CMV alone predicted no changes in the response to meningococcal polysaccharide. While CMV alone had no discernable effect on the antibody response to measles, the response of infants infected with both CMV and EBV was similar to that of infants infected with neither, suggesting that the effects of CMV infection countered the effects of EBV on measles antibody responses.

**Conclusions:**

The results of this exploratory study indicate that infection with EBV is associated with reduced antibody responses to polysaccharides and to measles vaccine, but suggest that the response to T-cell dependent antigens such as measles haemagglutinin may be restored by infection with CMV.

## Introduction

Infant vaccination is one of the most important strategies to combat infectious disease worldwide. However, it has been known for four decades that the efficacy of infant vaccines in Sub-Saharan Africa is lower than in high income settings [1] and that intercurrent infections like malaria may influence antibody responses [2], [3]. For instance, the efficacy of the live attenuated measles vaccine is typically over 90% in Europe and North America [4]–[6], but below 70% in West Africa [7]–[9].

In Sub-Saharan Africa, infection with the herpesviruses Epstein-Barr virus (EBV) and cytomegalovirus (CMV) usually occurs during infancy [10]–[12], after which they establish lifelong infection [13], [14]. Although infection is usually asymptomatic, both viruses have powerful effects on the lymphocyte populations involved in vaccine-mediated immunity. EBV infects B-cells and during acute infection, up to 50% of B-cells may be infected [15]. While EBV infection is usually asymptomatic in healthy individuals, it can cause severe disease in immunocompromised individuals and coupled with chromosomal translocations, causes Burkitt's lymphoma Burkitt's lymphoma in infants whose immune systems have been suppressed by malaria [16], [17]. In the absence of disease, EBV infected B-cells accumulate a relatively high number of mutations which suggests that EBV may influence the B-cell compartment even in the absence of clinical disease [18]. The effect of EBV infection on B-cell responses to vaccines or concurrent infections is unknown.

Unlike EBV, CMV has a powerful influence on T-cells even though T-cells are not a major target for CMV infection [19]. The T-cell populations of CMV-infected individuals show considerably higher levels of differentiation [20]–[23], even among young infants who are still receiving childhood vaccinations [24]. These effects vary with age as CMV-induced differentiation in the elderly is associated with reduced subpopulations of naïve T-cells and poor vaccine responses [23], [25], but infected infants show no such evidence of reduction of the naïve T-cell pool or of CMV-associated reduction in T-cell response to measles vaccine [26].

Polysaccharide vaccines stimulate B-cells independently of T-cells, suggesting that they may be particularly vulnerable to modulation by EBV. Although the meningococcus polysaccharide does not induce lasting immunity if administered before four years [27], the WHO still recommends vaccination irrespective of age to contain the outbreaks of meningococcal meningitis that periodically sweep the Sub-Saharan ‘meningitis belt’ [28], [29] and so it remains a valuable tool in child health.

By contrast, the live attenuated measles vaccine induces a broad range of T-cell and antibody responses [30], [31] so is unlikely to be so vulnerable to any one mechanism of modulation.

As early life CMV and EBV infection and relatively low vaccine efficacy are both characteristic of Sub-Saharan Africa, we hypothesised an association between CMV and EBV infection in infancy and reduced antibody responses to vaccines. We therefore quantified their influence on antibody responses to the polysaccharide vaccine against *Neisseria meningitidis* (meningococcus) and the live attenuated measles vaccine. We recruited infants from an ongoing cohort in a peri-urban area of The Gambia and administered the vaccines at nine months of age. Two months later, we compared the vaccine antibody responses of infants infected with CMV and/or EBV to those who remained uninfected.

## Materials and Methods

### Subjects and vaccinations

Infants were recruited at birth from the maternity ward of Sukuta Health Centre. Informed consent was obtained from their mothers and documented by signature or thumb print. Recruitment was restricted to healthy singletons, defined by a birth weight of at least 1.8kg and no congenital abnormalities, and whose mothers had not been admitted for hospital treatment during pregnancy or labour.

The area served by Sukuta Health Centre is peri-urban, and the population is characterised by low income and crowded accommodation. Breast feeding is usually continued well into the second year and 50% of infants are infected with CMV by 10 weeks of age [10]. The HIV status of study subjects was unknown, but adult prevalence in the region was below 2.5% at the time of the study [32], so is unlikely to be a significant confounder of this study. Exposure to malaria is low [33] and no cases of measles have been reported over the last 7 years. No children were clinically ill at the time of vaccination, and no outbreaks of measles, meningitis, Respiratory syncytial virus or Rotavirus occurred during the study.

All study subjects received childhood vaccinations according to the Expanded Program of Immunisation, which included inoculation with the live attenuated Edmonston-Zagreb strain of the measles virus (Serum Institute of India) at nine months. In addition, all infants were vaccinated with 50 µg each of meningococcus subtypes A and C capsular polysaccharide (Sanofi-Pasteur), also at nine months. All infants received a second dose of meningococcus conjugate C vaccine (kindly donated by Wyeth-Lederle) between three and four years of age to ensure continued protection.

The study was approved by the Gambia Government/MRC ethics committee.

### Sampling schedule

**Figure 1:**
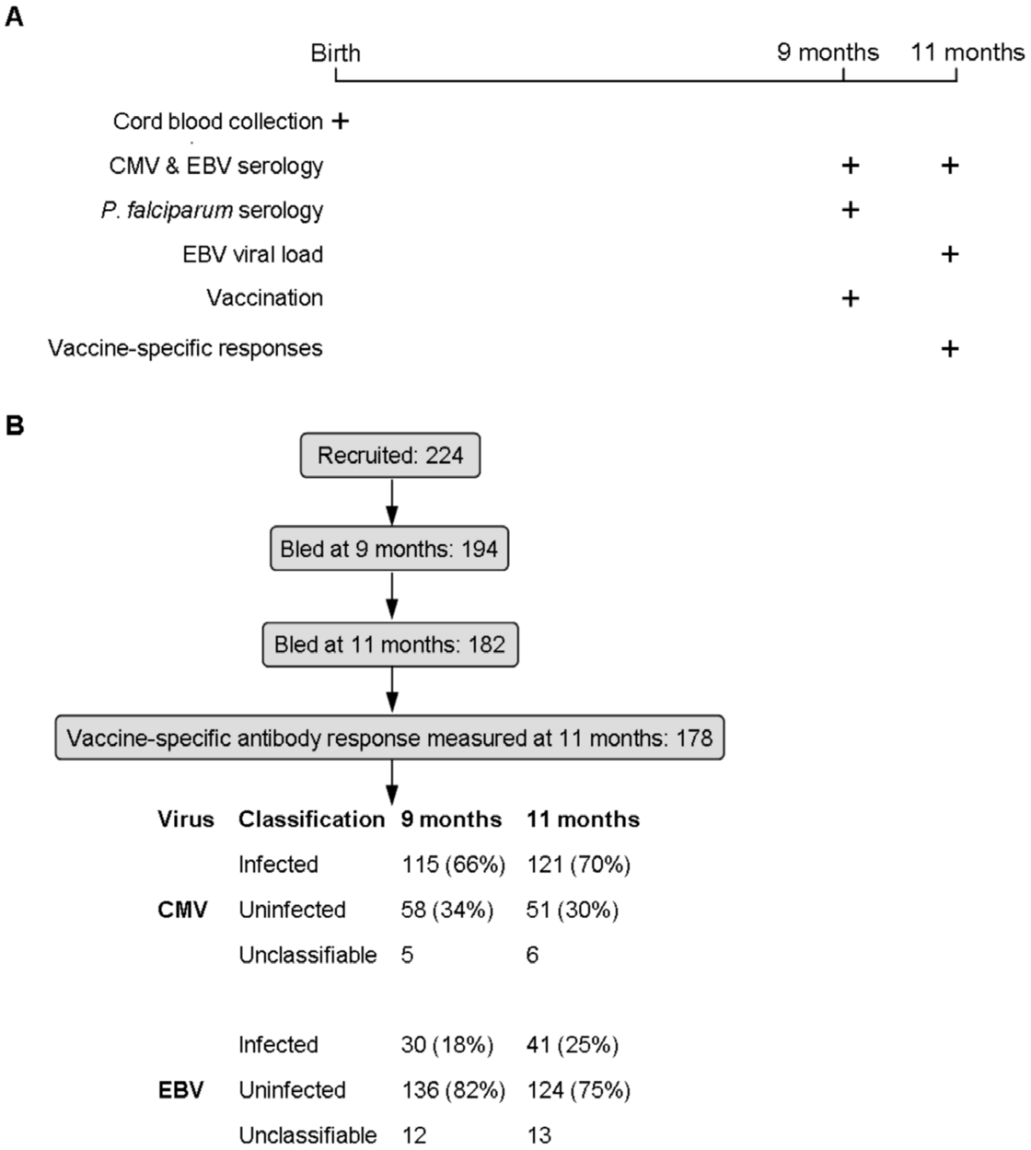
Study Design. **A** Study design showing times at which samples were collected, EBV and CMV serology was carried out, vaccines were administered and vaccine-specific responses were measured. **B** numbers of infants in the cohort and involved in analysis, and numbers infected with EBV and CMV at nine and eleven months.

Umbilical cord blood was collected from 224 infants at birth, and heparinised plasma was stored. At nine months, 1 ml of blood was collected into a serum separator tube (Becton-Dickinson) for serum collection from 194 infants. At eleven months, 4 ml of blood was collected for serum antibody measurements from 182 subjects and full laboratory data was available for 178 subjects (Fig. 1). Loss to follow–up was mainly due to migration (46%) or refusal to be bled (18%). All plasma and serum samples were stored at −50°C or below, and subjected to no more than three freeze-thaw cycles in the course of the study.

### Diagnosis of EBV and CMV

As many of the umbilical cord blood plasma samples contained substantial levels of maternally derived anti-EBV and CMV IgG, it was not possible to base diagnosis at nine or eleven months purely on IgG serostatus.

When infants received the measles and meningococcus vaccines at nine months, infants were classified as infected if they met one of two criteria, which were 1/virus-specific IgG levels exceeded the levels in their umbilical cord blood or 2/a detectable level of virus-specific IgM. Infants were classified as uninfected if they met one of three criteria: 1/virus-specific IgG and IgM levels were undetectable at the time of sampling, 2/virus-specific IgG and IgM levels were both undetectable at eleven months, as infants infected at nine months would have an antibody response by eleven months, or 3/virus-specific IgG levels were detectable but below cord blood levels at nine months while IgM levels converted from undetectable to detectable between nine and eleven months.

Two months after receiving the measles and meningococcus vaccines, at eleven months of age, infants were classified as infected if they met one of three criteria: 1/virus-specific IgG levels exceeded the levels in their umbilical cord blood, 2/if they had detectable levels of virus-specific IgM, or 3/if they had been classified as infected at nine months. Infants were classified as uninfected if they met one of three criteria: 1/virus-specific IgG and IgM levels were both undetectable at nine months and IgM levels remained undetectable at eleven months, or 2/virus-specific IgG and IgM levels were both undetectable at eleven months.

Infants for whom none of the above criteria could be met were unclassified.

Virus-specific antibody levels were measured using ETI-VCA-G and ETI-EBV-M Reverse ELISAs (Diasorin), which detect IgG and IgM respectively to the viral capsid antigen (VCA). Infection with CMV was diagnosed using the ELICYTOK-G Plus ELISA (Diasorin) to detect IgG and either the ETI-CYTOK-M Reverse Plus (Diasorin) or the Cytomegalovirus IgM CAPTIA™ ELISA (Trinity Biotech) to detect IgM.

### Diagnosis for exposure to malaria parasites

Exposure to malaria was assessed by testing for the presence of IgG antibody specific for two merozoite surface antigens, apical membrane antigen 1 (AMA1) [34] and the two allelic types of merozoite surface antigen 2 (MSP2) [35] using an ELISA method as previously described [36]. Samples were classified as seropositive if the optical density reading was above 3 standard deviations of the mean value obtained by testing 20 sera from non-immune tourists visiting The Gambia [33].

### Measurement of vaccine responses

Antibody responses to measles and meningococcus were measured using the serum samples collected at eleven months of age. Responses to meningococcus were quantified with an ELISA adapted from a previously published protocols [37]. Standards were prepared from reference serum CDC 1992 (NIBSC) at ^1^/_100_ for meningococcus A and ^1^/_200_ for meningococcus C, and eight doubling dilutions of triplicate preparations were carried out for each microplate, enabling results to be expressed as µg ml^−1^.

Anti-measles antibody response was measured using the haemagglutinin antibody inhibition assay (HAI) [38], and calibrated against the second WHO standard. Assay sensitivity was 15.6 mIU and minimum detectable value was 31.2 mIU. Results are expressed as the log_2_ titre at which no haemagglutination was observed.

### Assessment of EBV viral load

Samples were collected from 40 of the 43 EBV-seropositive infants at eleven months of age. Aliquots of 200 µl of blood were centrifuged at 2000 *g* for 5 min, plasma was removed and DNA was isolated into 50 µl using the DNA Mini Kit reagents (Qiagen). Positive control EBV DNA was extracted from B95-8 cells [39] and Namalwa cells [40] using similar methods. The DNA concentration of the samples was determined by UV spectrophotometry. The number of EBV genome copies in the DNA samples was determined by real-time PCR. Briefly, 5 µl of DNA (1–5 ng/µl) was included in a 25 µl reaction using the manufacturer's protocol (Qiagen Quantitect Virus kit). After a 10 min step at 95°C step to activate the polymerase, cycling (40 cycles) was performed. Cycling (40 repeats) (15 s at 95°C, followed by 60 s at 60°C with data acquisition at either 470 nm source, 510 nm detection for FAM (EBV BALF5 probe) or 530 nm source, 555 nm detection for JOE (B2M probe). The primers and probes are described in Table 1. The EBV genome number in test samples was quantified relative to B95-8 and Namalwa DNA and the EBV copy number per cell in the test samples was expressed relative to the β-2 microglobulin gene [41]. The lower limit of detection was four EBV copies in 5 µl DNA sample ( = 50 µl original blood volume).

**Table 1:** Primers used for quantification of EBV by real-time PCR.

Name	Function	Sequence (5′ to 3′)
mo 052	EBV BALF5 forward primer	CCTTTGGCGCGGATCCTC
mo 053	EBV BALF5 reverse primer	TCCTTCTTGGCTAGTCTGTTGAC
mo 054	EBV BALF5 probe5′ 6-FAM, 3′ TAMRA	CATCAAGAAGCTGCTGGCGGCC
mo 055	B2M forward primer	GGGAATTGATTTGGGAGAGCATC
mo 056	B2M reverse primer	AGGTCCTGGCTCTACAATTTACTAA
mo 057	B2M probe5′ HEX, 3′ TAMRA	AGTGTGACTGGGCAGATCATCCACCTTC

### Statistical analysis

Association for infection with CMV and EBV was quantified using Pearson's chi-square test. Differences in the log_10_ transformed vaccine response between CMV and EBV infection status (and their interaction) were tested using linear regression adjusting for the possible confounding effect of malaria infection. Due to the high proportion of zero IgM responses to meningococcus C, the responses were dichotomised around 0.10 µg ml-1and analysed using logistic regression.

The HAI titres were categorized into 8 groups and ordinal logistic regression was used to study the association with EBV and CMV infections status, allowing for the possible confounding effect of pre-vaccination measles antibody.

As the meningococcal ELISAs generated continuous data, they were compared to EBV viral load by ANCOVA using CMV serostatus as a fixed factor. If CMV serostatus was non-significant, EBV viral load was compared to the antibody level by Spearman's correlation coefficient, and the four derived significances were corrected for multiplicity by the step-down Bonferroni method.

Linear regression was used to test for the association between EBV viral load and anti-meningococcus IgG and IgM adjusting for CMV serostatus.

Analyses were performed using Stata 11 (Statacorp) and Matlab 7.4 (The MathWorks Inc). Differences were considered significant at p<0.05.

## Results

Analyses were restricted to the 178 subjects who were bled and vaccinated at 9 months of age and who had antibody data at 11 months of age.

### More infants were infected with CMV than EBV

CMV infection was usually earlier than EBV (Fig. 1). At nine months, 115 of 173 (66%) classifiable subjects were CMV-infected as opposed to 30 of 166 EBV-infected subjects. 10% of the CMV uninfected subjects and 8% of the EBV uninfected subjects were infected between 9 and 11 months of age. No significant associations between seropositivity for CMV and EBV were found.

### Few infants had been exposed to malaria or measles

At nine months, 30 of 176 (17%) samples tested were seropositive for malaria. Seropositivity for malaria was not associated with seropositivity for CMV or EBV.

Seropositivity for measles was uncommon before vaccination, with only six of 178 (3.4%) infants tested immediately before vaccination having a detectable level of haemagglutinating antibody activity.

### EBV infection was associated with low antibody responses to meningococcus polysaccharide

There were no significant statistical interactions between the effects of EBV and CMV on any of the responses to meningococcus measured.

Levels of IgG and IgM to meningococcus A and C at 11 months were compared by EBV serostatus at the time the vaccine was administered at nine months. IgG responses were significantly lower in EBV^+^ infants (p<0.05). However, there were no detectable differences between EBV^+^ and EBV^−^ infants in the anti-meningococcus A or C IgM levels (Table 2). When levels of IgG and IgM to meningococcus A and C were compared by EBV serostatus at the time of sampling at eleven months, both IgG and IgM concentrations were lower in EBV^+^ infants (p<0.01) (Table 3).

**Table 2:** Antibody responses to meningococcus A and C, grouped by EBV/CMV serostatus at nine months.

Meningococcus strain	Antibody isotype	Group	n	Median (µg ml^−1^)	IQR	P*
A	IgG	EBV^−^	136	2.05	1.30–3.92	**0.03**
A	IgG	EBV^+^	30	1.40	0.80–2.31	
A	IgM	EBV^−^	136	1.29	0.68–2.33	0.10
A	IgM	EBV^+^	30	0.97	0.35–1.74	
C	IgG	EBV^−^	136	3.20	1.59–6.80	**0.04**
C	IgG	EBV^+^	30	2.47	1.21–3.86	
C	IgM	EBV^−^	136	0.10	0.01–0.19	0.08
C	IgM	EBV^+^	30	0.08	0.02–0.12	
A	IgG	CMV^−^	58	2.00	1.40–4.41	0.12
A	IgG	CMV^+^	115	1.75	1.16–3.56	
A	IgM	CMV^−^	58	1.30	0.69–2.36	0.55
A	IgM	CMV^+^	115	1.13	0.58–2.27	
C	IgG	CMV^−^	58	3.67	1.77–6.63	0.28
C	IgG	CMV^+^	115	2.68	1.35–5.63	
C	IgM	CMV^−^	58	0.09	0.01–0.19	0.83
C	IgM	CMV^+^	115	0.09	0.02–0.17	

*Calculated by linear regression model. Significant values in bold.

Infection with EBV at time of vaccine administration at nine months predicts reduced antibody responses to both meningococcus A and C, but infection with CMV has no effect. Groups refer to the CMV and EBV serostatus at the time the vaccine was administered at nine months.

Infection with CMV at either time of vaccination or at time of sampling was not associated with anti-meningococcus antibody levels at 11 months (Table 2, Table 3), and none of the responses correlated with EBV viraemia at 11 months of age (data not shown).

**Table 3:** Antibody responses to meningococcus A and C, grouped by EBV/CMV serostatus at eleven months.

Meningococcus strain	Antibody isotype	Group	n	Median (µg ml^−1^)	IQR	P*
A	IgG	EBV^−^	124	2.15	1.35–4.13	**0.003**
A	IgG	EBV^+^	41	1.43	0.90–2.31	
A	IgM	EBV^−^	124	1.38	0.71–2.44	**0.002**
A	IgM	EBV^+^	41	0.92	0.32–1.30	
C	IgG	EBV^−^	124	3.44	1.75–7.44	**0.006**
C	IgG	EBV^+^	41	2.41	1.19–3.80	
C	IgM	EBV^−^	124	0.11	0.02–0.19	**0.01**
C	IgM	EBV^+^	41	0.07	0.00–0.12	
A	IgG	CMV^−^	51	2.03	1.31–4.44	0.08
A	IgG	CMV^+^	121	1.86	1.20–3.56	
A	IgM	CMV^−^	51	1.35	0.67–2.46	0.39
A	IgM	CMV^+^	121	1.13	0.61–2.25	
C	IgG	CMV^−^	51	3.61	1.95–6.19	0.26
C	IgG	CMV^+^	121	2.70	1.37–6.18	
C	IgM	CMV^−^	51	0.09	0.01–0.19	0.86
C	IgM	CMV^+^	121	0.09	0.02–0.17	

*Calculated by linear regression model. Significant values in bold.

Infection with EBV at eleven months predicts reduced antibody responses to both meningococcus A and C, but infection with CMV has no effect. Groups refer to EBV and CMV serostatus at the time of sampling at 11 months.

Following these initial analyses, we then performed several tests of the robustness of our analytic approach.

The majority of subjects who could not be classified had detectable levels of IgG to CMV or EBV, but at levels lower than in the umbilical cord blood. In order to test whether the results could have been biased by failure to classify infants that were actually uninfected, we repeated the regression analysis with all unclassified infants as uninfected, and found that the associations still remained significant (data not shown).

Eleven infants seroconverted for EBV between 9 and 11 months, and six seroconverted for CMV though none seroconverted for both To establish whether the difference between EBV-infected and uninfected infants was due to the effect of infection between the time of vaccination and sampling, we repeated the regression analysis excluding any infants that seroconverted between the two sampling times. The significant associations remained the same as in the original analysis (data not shown).

To test whether malaria infection had any effect on anti-meningococcus antibody levels, we repeated the regression analysis with seropositivity to *P. falciparum*, defined as a detectable response to any of the three antigens tested, as a factor. We found no effect on the associations (data not shown).

### EBV infection predicted low antibody responses to measles unless there was concurrent CMV infection

There were significant interactions between the effects of EBV and CMV infection on the anti-measles antibody response, so it was inappropriate to perform multiple comparisons on the four possible groups.

**Figure 2:**
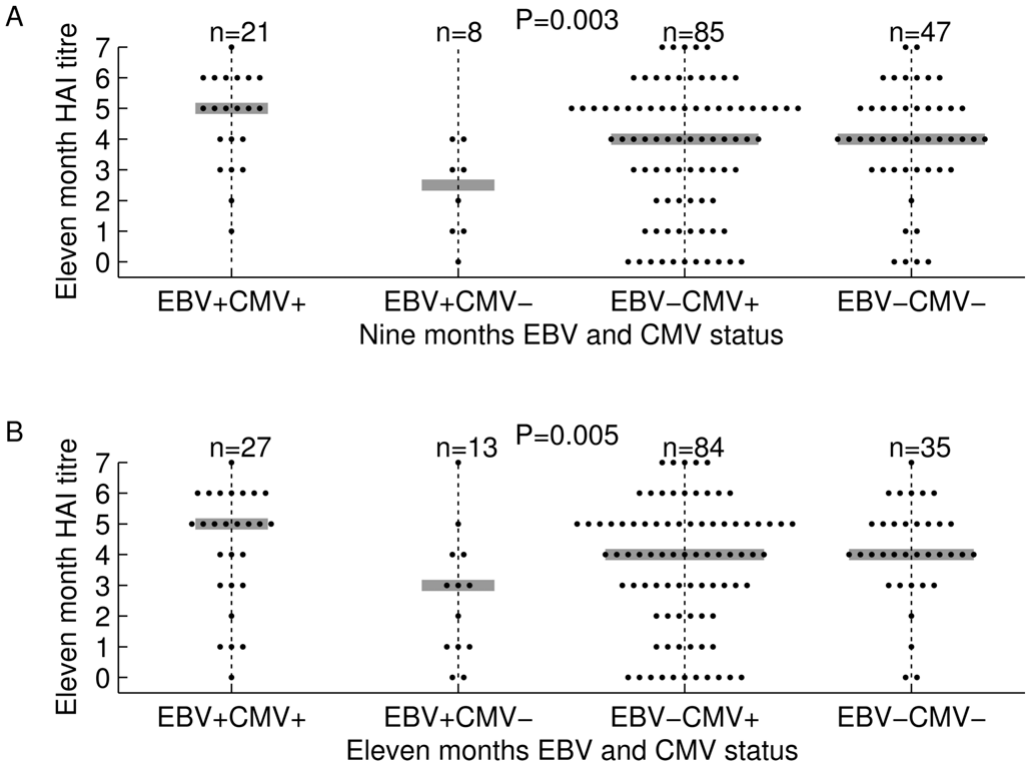
Infection with EBV but not CMV downregulates antibody responses to measles. Infection with EBV is associated with reduced antibody responses to measles unless infants are coinfected with CMV. Plots of serum haemagglutinin-inhibiting activity at eleven months of age, plotted against the serostatus at **A** the time of vaccination at nine months of age and **B** the time of sampling at eleven months of age. Titres are expressed as log_2_. Grey bars indicate medians. Significances refer to the statistical interaction between the effects of EBV and CMV infection.

The same trends were apparent whether the infants were classified by CMV and EBV serostatus at the time of vaccination at nine months or the time of antibody measurement at eleven months. In both cases, antibody levels of the EBV^+^CMV^−^ infants were considerably lower than those of any of the other three groups, while differences between the other three groups were small (Fig. 2).

The EBV^+^CMV^−^ group accounted for only 8 of the 161 (5.0%) classifiable infants at nine months and 13 of 159 (8.2%) at eleven months. The median log (base 2) HAI titre of the EBV^+^CMV^−^ infants was 2.5 as compared to 4.0 among EBV^−^CMV^−^ infants classified by serostatus at nine months. The difference based on serostatus at eleven months was smaller, but the median inhibiting titre was 3.0 among EBV^+^CMV^−^ infants compared to 4.0 among EBV^−^CMV^−^ infants.

CMV infection alone showed little effect on antibody titre, but appeared to offset the reduced antibody response associated with EBV as the median titre among EBV^+^CMV^+^ infants was 5.0 both at nine and eleven months (Fig. 2).

Incorporating pre-vaccination measles antibody titre into the regression model had no effect on the associations between CMV and EBV with anti-measles antibodies, or the interaction between CMV and EBV, and neither the presence nor the titre of pre-vaccination anti-measles antibodies was associated with anti-measles antibodies in any of the groups at 11 months.

## Discussion

We found that EBV infection prior to or shortly after vaccination at 9 months of age predicted lower antibody responses to both the T-cell-dependent measles and T-cell-independent meningococcus polysaccharide antigens, but that co-infection with CMV was associated with responses equivalent to those found in uninfected infants.

We can only speculate on the immune mechanisms underlying these interactions as the study focussed on antibody measurements. However several mechanisms of interaction between viral infections have been described. For instance, one virus may induce an immune response that inhibits growth of another [42], [43], or specific immune responses induced by one virus may cross-react with epitopes expressed by another [44].

The lower antibody responses in EBV-infected infants are consistent with the finding that EBV infection of B-cells induces mutations that may interfere with antibody production [18]. Work on mouse models showed that the immune system can only support a finite number of antibody producing cells [45], so it is also possible that EBV-induced expansion reduces the available niches for vaccine-specific antibody producing cells.

Co-infection with CMV appeared to return antibody levels to the T-cell-dependent haemmaglutinin to levels found in EBV^−^ infants. As there was no effect of CMV infection on antibody to the T-cell-independent meningococcus vaccine, this suggests restoration was mediated through T-cells. In The Gambia, we have previously found that CMV infection drives CD4 T-cell differentiation in infants and that cellular responses to CMV correlate with the antibody response to measles vaccine [26], which suggests that exposure to CMV may enhance antibody production through non-specific upregulation of CD4 T-cell mediated help. Studies in The Gambia [26] and Malawi [46] have shown that CMV infection is associated with a relatively high proportion of memory cells in both the CD4 and CD8 T-cell compartments, which accords with our findings. These putative interactions of CMV with vaccines need to be studied on a larger scale in early infancy when the incidence of CMV is highest and vaccination most intense.

We were unable to distinguish the effects of infection with EBV and CMV at time of vaccination from the effects at time of sampling so could not establish whether low vaccine antibody levels in EBV-infected infants are programmed at the time of infection or whether infections after vaccination are able to down regulate current antibody production. In either case, approximately 20% of infants were infected with EBV at nine months and consequently subject to lower immune response which is a substantial number, even if post-vaccination EBV infection does not have the same downregulatory effects.

It is unlikely that EBV infection alone is sufficient explanation for reduced efficacy of measles vaccine in Africa, as only 10% of infants were EBV^+^CMV^−^. However the magnitude of the reduction in antibody responses suggests that EBV may be a contributing factor along with early age of immunisation, persistence of maternal antibody [47] and intense exposure during measles outbreaks due to overcrowding [48].

Malaria has been associated with immunosuppression of responses to viral infection [17], [49] and the meningococcal polysaccharide and *Haemophilus influenzae* vaccines [2], [3]. However the lack of association between low level malaria exposure and CMV or EBV infection in this cohort, or between malaria exposure and antibody response to vaccines makes it unlikely that malaria infection confounded the association between antibody levels and CMV and EBV infection in this cohort. Together with the low HIV prevalence and lack of concurrent disease outbreaks, it is unlikely that another infection was behind the observed differences. Due to shortage of sera we could not measure pre-vaccination meningococcal antibody concentrations. However, previous studies in the region have shown that maternal antibody decays quickly and that infants have undetectable or very low levels [50] and very low carriage rates [51], so past or intercurrent infection with meningococci are unlikely to have influenced our results.

Our exploratory study was relatively limited in size and cannot provide a mechanistic explanation for the potentially important findings. Larger studies will be necessary to establish whether CMV or EBV infection bears on vaccine efficacy or enhances susceptibility to invasion by polysaccharide-encapsulated bacteria such as *Streptococcus pneumoniae* or *Neisseria meningitidis*, which are common in this region. Furthermore, understanding the interaction of these common early life infections with the infant immune system may shed light on the non-specific effects of vaccines reported in West Africa [52], [53].

### Conclusions

Infection with EBV reduced antibody responses to the T-cell independent meningococcal polysaccharide vaccines while infection with CMV had no effect. Infection with EBV alone reduced the antibody response to the live measles vaccine, but co-infection with CMV reversed the effects of EBV and elevated the antibody response to levels similar to those of infants infected with neither virus.
